# Acute effect of passive cycle-ergometry and functional electrical stimulation on nitrosative stress and inflammatory cytokines in mechanically ventilated critically ill patients: a randomized controlled trial

**DOI:** 10.1590/1414-431X20208770

**Published:** 2020-04-09

**Authors:** E.E.T. França, J.P.V. Gomes, J.M.B. De Lira, T.C.N. Amaral, A.F. Vilaça, M.D.S. Paiva, U.F. Elihimas, M.A.V. Correia, L.A. Forgiarini, M.J.C. Costa, M.A. Andrade, L.C. Ribeiro, C.M.M.B. De Castro

**Affiliations:** 1 Departamento de Fisioterapia e Programa de Pós-graduação em Fisioterapia, Universidade Federal da Paraíba, João Pessoa, PB, Brasil; 2 Unidade de Terapia Intensiva, Hospital Agamenon Magalhães, Recife, PE, Brasil; 3 Programa de Residência de Clínica Médica, Hospital Otávio de Freitas, Recife, PE, Brasil; 4 Programa de Pós-graduação em Educação Física e Hebiatria, Universidade de Pernambuco, Recife, PE, Brasil; 5 Departamento de Fisioterapia e Programa de Pós-graduação em Saúde e Desenvolvimento Humano, Universidade La Salle, Canoas, RS, Brasil; 6 Departamento de Fisioterapia, Universidade Federal de Pernambuco, Recife, PE, Brasil; 7 Departamento de Fisioterapia e Medicina Tropical, Universidade Federal de Pernambuco, Recife, PE, Brasil

**Keywords:** Cytokines, Nitrosative stress, Intensive care unit, Physiotherapy

## Abstract

Early mobilization is beneficial for critically ill patients because it reduces muscle weakness acquired in intensive care units. The objective of this study was to assess the effect of functional electrical stimulation (FES) and passive cycle ergometry (PCE) on the nitrous stress and inflammatory cytometry in critically ill patients. This was a controlled, randomized, open clinical trial carried out in a 16-bed intensive care unit. The patients were randomized into four groups: Control group (n=10), did not undergo any therapeutic intervention during the study; PCE group (n=9), lower-limb PCE for 30 cycles/min for 20 min; FES group (n=9), electrical stimulation of quadriceps muscle for 20 min; and FES with PCE group (n=7), patients underwent PCE and FES, with their order determined randomly. The serum levels of nitric oxide, tumor necrosis factor alpha, interferon gamma, and interleukins 6 and 10 were analyzed before and after the intervention. There were no differences in clinical or demographic characteristics between the groups. The results revealed reduced nitric oxide concentrations one hour after using PCE (P<0.001) and FES (P<0.05), thereby indicating that these therapies may reduce cellular nitrosative stress when applied separately. Tumor necrosis factor alpha levels were reduced after the PCE intervention (P=0.049). PCE and FES reduced nitric oxide levels, demonstrating beneficial effects on the reduction of nitrosative stress. PCE was the only treatment that reduced the tumor necrosis factor alpha concentration.

## Introduction

Early mobilization is beneficial for critically ill patients because it reduces muscle weakness acquired in intensive care units (ICU). Mechanical ventilation and immobility are the main risk factors for developing muscle alterations, and knowledge regarding these risks and their identification are very important (1). This dysfunction is associated with inflammatory deregulation that apparently contributes to the onset of myopathy. The mechanism of muscle loss due to immobility has not been fully clarified to date, and the synergism among oxidative and nitrosative stress, inflammatory cytokines, and immobility presumably causes or accelerates muscle atrophy (2).

Important results regarding the rehabilitation of critically ill patients have been published in the literature, showing the benefit of increasing the practice of rehabilitation exercises, which aim to reduce symptoms and improve function regardless of disease stage (4). Physical exercise guidelines for critically ill patients currently exist. These guidelines are based on levels of mobility ranging from passive exercise to walking in the ICU (5).

According to these levels of mobility, both passive cycle-ergometry (PCE) and functional electrical stimulation (FES) application provide muscle contraction without increasing cardiovascular work, thus allowing patients with severe ventilatory insufficiency to tolerate this type of peripheral muscle training (6).

The training aims to improve the function of muscle properties, intramuscular blood flow, maximal strength production, and endurance through repeated contractions, thereby preventing muscle atrophy (7). Theoretically, the higher the intensity of the exercise the greater is the increase in cardiac output and maximal oxygen uptake in response to the increase in muscle oxygen consumption (8,9). This increase in metabolism during exercise also occurs in critically ill patients (10). To our knowledge, muscle and cardiovascular responses to different forms of bed exercise have never been compared. The most commonly used types of bed exercise are PCE and quadriceps electrical stimulation (6). However, their effects on immune response parameters and nitrosative stress in these patients remain unclear.

The present study aimed to assess the acute effect of lower limb PCE and quadriceps FES on nitrosative stress and inflammatory cytokines in critically ill patients on mechanical ventilation (MV).

## Material and Methods

### Design

This was a controlled, randomized, open clinical trial carried out in a 16-bed ICU between December 2013 to February 2016. The study was approved by the institutional review board (Research Ethics Committee of the Agamenon Magalhães Hospital). In conformity with the Declaration of Helsinki, written informed consent to participate in the study was required from all patients. When consent was given by a proxy, the patient was informed as soon as possible and written consent was obtained. The published study protocol (trial registration: ReBEC, RBR-6pxpx9) complied with the Consolidated Standards of Reporting Trials (CONSORT) guidelines for clinical trials. The randomization of the patients was performed through the website <http://www.randomization.com>.

### Patients

The inclusion criteria were patients older than 21 years, intubated for at least 24 h, under MV with adequate cardiac reserve (demonstrated by variability <20% heart rate at rest), systolic blood pressure between 90 and 180 mmHg, normal electrocardiogram, peripheral capillary oxygen saturation >90%, fraction of inspired oxygen <60%, respiratory rate <25 bpm, hemoglobin >7 g/dL, platelets >20,000 cells/mm^3^, and without sepsis diagnosis. Patients were excluded if they were unable to walk without assistance before ICU admission, pregnant, with a body mass index >35 kg/m^2^, previously diagnosed with neuromuscular and vascular disease, with a history of stroke, with skin lesions at the electrode attachment site, with unconsolidated fracture, or with a definitive pacemaker. Patients with signs of low or high blood pressure or those in whom venous access was obstructed by a clot that prevented blood collection were removed from the study.

### Interventions

**Figure 1:**
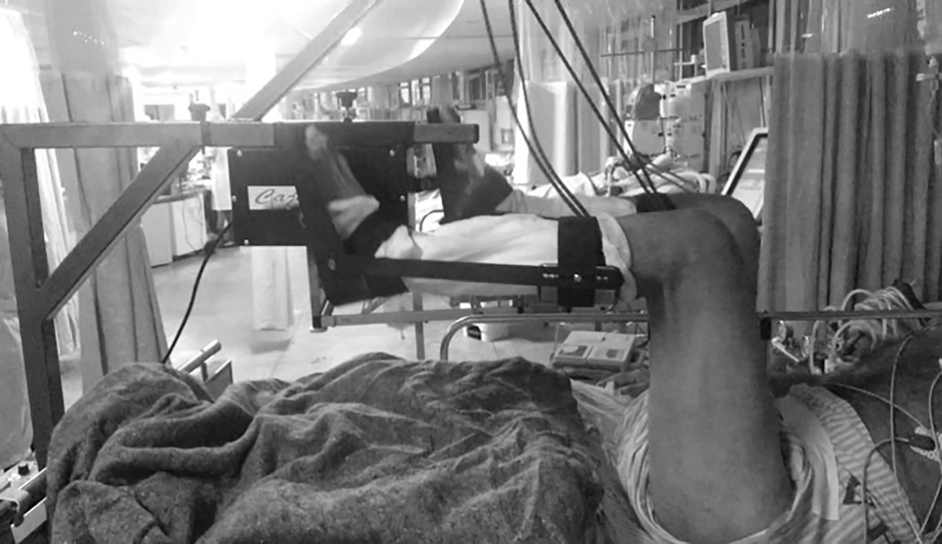
Illustration of the application of lower limb passive cycle-ergometry in critically ill patients under mechanical ventilation.

The initial evaluation was based on medical records, clinical history, diagnosis, and use of vasoactive drugs and sedatives. The order of the interventions was randomized to one of the 4 groups using a Latin square design. The control group underwent no therapeutic intervention during the study period and were subject to physiotherapy at the ICU in other periods. The lower limb PCE group performed lower limb ergometry using the cycle ergometer (Flex Motor with sensor; Cajumoro, Brazil) with 30 rotations programmed per min for 20 min, according to the protocol of França et al. (11) (Figure 1).

**Figure 2:**
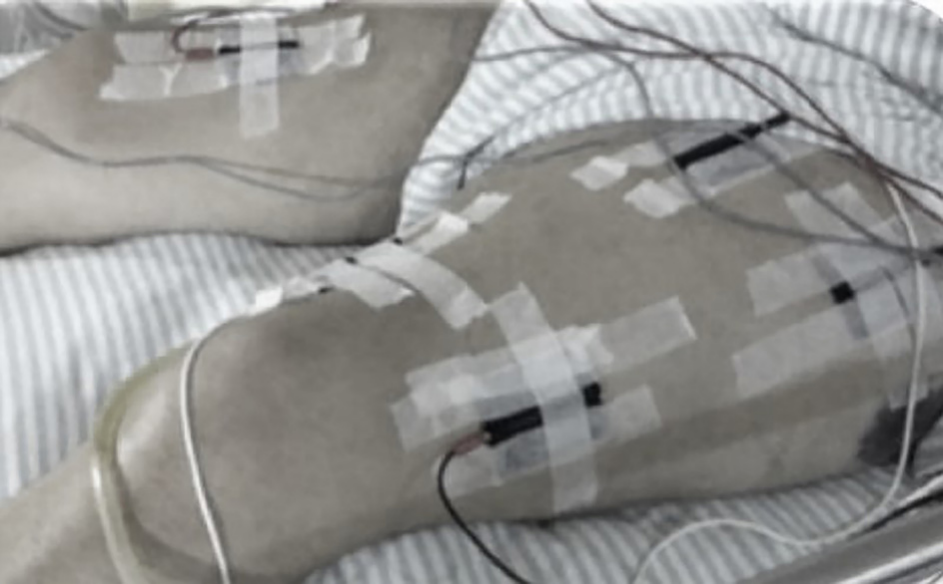
Illustration of the application of functional electrical stimulation to the quadriceps muscles of critically ill patients under mechanical ventilation. Quadriceps involve rectus femoris and vastus lateralis muscles.

The patients randomized to the lower-limb FES group were subjected to functional electrical stimulation of the rectus femoris and vastus lateralis muscles of the quadriceps. The FES (Neurodyn-4 channel, Inbramed, Brazil) was programmed with a 500 µs pulse width, 50 Hz frequency, 2 s of elevation, 5 s of support, and 2 s of descent with a 1:1 ON/OFF time. Current intensity was set by visualization of muscle contraction. If this visualization was not possible, then it was set by palpation of the stimulated muscle. The session lasted 20 min for each quadriceps muscle stimulated, and FES was performed simultaneously in the quadriceps of both lower limbs (Figure 2) (12). Finally, in the fourth group, patients underwent both interventions, lower limb PCE and FES, with their order determined randomly through Latin square design, totaling a time of 40 min of therapy.

### Evaluation

All therapies applied to the four study groups were performed in a single setting because our objective was to assess the acute effect of those physiotherapeutic interventions applied to critically ill patients. Thus, after including the patient in the study, the first blood sample was collected immediately before the protocol was started in both groups, and the second blood collection was performed one hour after completion of the therapeutic intervention. 

According to well-established methods by França et al. (11), the blood was stored and transported in three 4-mL vacutainer blood collection tubes (Vacutainer¯, Brazil) containing dipotassium ethylenediamine tetraacetic acid in a refrigerated container to the Laboratório de Imunologia Keizo Asami, Federal University of Pernambuco. The blood samples were diluted in 10-mL Falcon tubes for nitrosative stress analysis by determining nitric oxide (NO) production in cells (monocytes) and assessing immune response parameters, including cytokines tumor necrosis factor (TNF)-α, interferon gamma (IFN-γ), interleukin 6 (IL-6), and interleukin 10 (IL-10), which were assessed by plasma count (11).

Monocytes were obtained from peripheral blood and then diluted at a 1:2 ratio in sterile phosphate-buffered saline (PBS) at room temperature (22 to 25°C; 10 mL blood+10 mL PBS). Then, 10 mL of Histopaque (1077, Sigma, USA) was added to 20 mL of the suspension, and the entire content was centrifuged for 30 min at 1,600 *g* (25°C). Immediately thereafter, plasma was aspirated. The layer formed by peripheral blood mononuclear cells was collected and transferred to another test tube. The same volume of PBS aspirate was added, and the sample was centrifuged for 15 min under the conditions described above. The supernatant was discarded, and the sediment was resuspended in 1 mL of complete Roswell Park Memorial Institute 1640 culture medium (Thermo Fisher Scientific, Gibco^TM^, Brazil) containing 3% fetal bovine serum and antibiotics (100 U/mL penicillin and 100 µg/mL streptomycin). The count was performed by placing an aliquot of the suspension in a Neubauer chamber and adding trypan blue dye at a 1:10 ratio. This dye was used to count cells and assess their viability. Based on the count, the concentration of 1×10^6^ cells was standardized for each mL of culture medium (11).

As described previously by França et al. (11), NO production was assessed in cultured monocytes treated with *E. coli* lipopolysaccharide (LPS). These cells were stimulated (positive control (C+)) or non-stimulated (negative control (C-)). In each group, the concentration was adjusted to 1×10^6^ cells in 1 mL of culture medium in each well of the plate. Subsequently, the cells were treated at a dose of 10 µg/mL LPS for 24 h. NO release was assessed using the GRIESS method by adding 50 µL of the reagent GRIESS (1 g of sulphanilamide, Sigma 9251; 0.1 g of N-(naphthyl) ethylenediamine dihydrochloride, Sigma 5889; 2.5 mL of phosphoric acid, and distilled water to 100 mL). The plate was incubated for 10 min in the dark. The readings were performed at 540 nm in an enzyme-linked immunosorbent assay (ELISA) plate reader (Dynatech MR 5000, Brazil). The sensitivity threshold of the assay was 1.56 µM (11).

Conversely, the serum levels of IL-6, IL-10, TNF-α, and IFN- γ were determined by ELISA using commercial ELISA kits for IL-6, IL-10, IFN-γ (Invitrogen, USA), and TNF-α (BioSource¯, TNF-α EASIA, Belgium) according to manufacturer instructions. The reading was performed in a plate reader (BioRad, Japan) at 450 nm and compared with a standard curve constructed with known concentrations of the recombinant mediators (11).

### Sample size and statistical analysis

Due to the difficulty of finding studies that evaluated the alteration of the inflammatory profile and nitro-oxidative excess in critically ill patients using FES and PCE, the sample size calculation was idealized and scaled to consider the frequency of use of one of these protocols in the year prior to the collection in the hospital. Based on these considerations, a survey of medical records identified that out of every 100 inpatients, only two performed FES or PCE.

**Figure 3:**
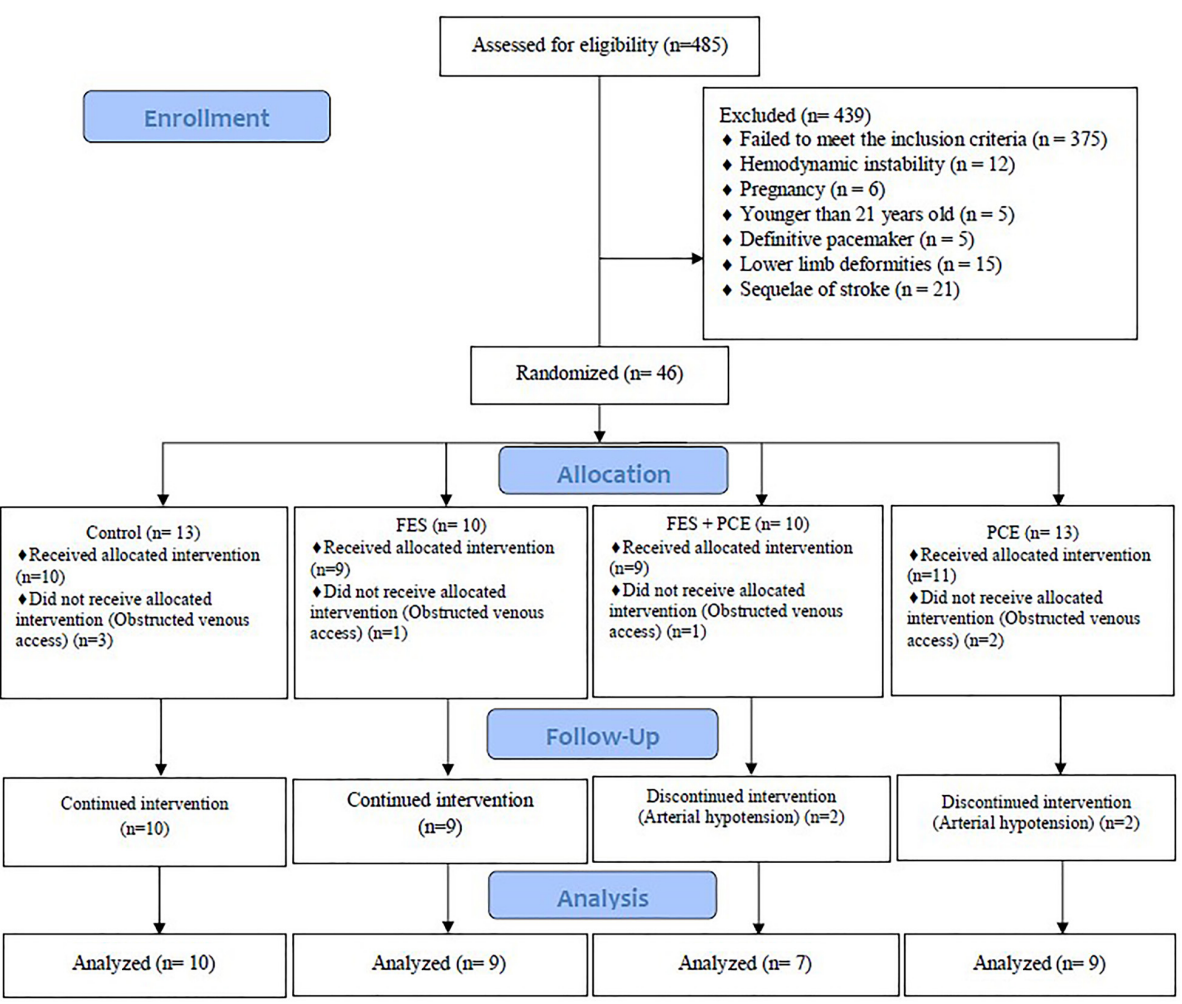
Study design. FES: functional electrical stimulation; PCE: passive cycle-ergometry.

The WinPepi program (PEPI-for-Windows) (13) was used to quantify the sample size according to the following criteria: confidence interval of 95%; sampling error of five percentage points; estimated prevalence of 2% (total number of patients admitted to the ICU who did neuromuscular electrical stimulation or passive mobilization with the lower limb cycle ergometer (14)), and sample loss of 35%, therefore totaling a minimum sample size of 47 patients. The patients were chosen by drawing lots after randomization carried out using the WinPepi program. From this calculation, patients were allocated to the four groups evaluated (control, PCE, FES + PCE, and FES) (Figure 3) from the randomization process.

Continuous data are reported as means±SD or median and 25–75% percentile, whereas the absolute data are reported as total value and percentage. The Kolmogorov-Smirnov test was used to test the normality of the study variables. The comparative analyses between the four groups were performed using analysis of variance (ANOVA) with the Bonferroni *post*-test. The non-parametric Wilcoxon signed-rank test was used to compare the results obtained before and after the study protocol in each group. The significance level was set at 5%. Data were analyzed using GraphPad Prism 4 (USA).

## Results

A total of 46 patients were included in the study. However, only 35 patients completed the therapy. These patients were distributed as follows: control group (n=10), FES group (n=9), PCE group (n=9), and PCE + FES group (n=7) (Figure 3).

The demographic characteristics and clinical parameters are outlined in Table 1. The mean age of the patients was 61.03±17.75 years and 62.85% were women. The mean acute physiology and chronic health evaluation (APACHE II) score was 21.97±5.55. The median time of mechanical ventilation was 5 days (interquartile range: 3 days). The most common reasons for ICU hospitalization included respiratory tract infection, decompensated heart failure, and urinary tract infection. No significant differences between groups were identified (Table 1). No differences in demographic and clinical laboratory characteristics of the patients were found in the four study groups, demonstrating the homogeneity of the study groups for all parameters tested.

**Table 1:** Demographic and clinical variables, admission motives, and comorbidities in the four groups studied.

Variable	Groups				
	Control (n=10)	FES (n=9)	PCE + FES (n=7)	PCE (n=9)	P value
Age (year)	56.80±12.80	64.11±18.19	64.19±10.54	60.11±27.15	0.800
Height (cm)	164.70±8.82	161.78±6.26	166.71±6.42	158.56±6.32	0.126
Weight (kg)	71.10±13.37	66.67±9.35	68.57±14.53	68.89±11.79	0.889
BMI (kg/cm^2^)	25.92±4.02	26.19±3.29	29.88±13.5	27.35±5.48	0.341
APACHE II	22.60±4.22	20.78±5.58	21.71±3.86	22.67±8.36	0.888
WB-24 h (mL)	972.20±703.84	993.78±551.04	1504.14±944.82	838.33±693.26	0.311
RASS	-4.50±0.707	-4.44±0.726	-4.29±0.951	-4.89±0.333	0.098
T MV (days)	4.9±2.80	5.67±3.35	4.29±1.38	6.44±3.64	0.174
T ICU (days)	4.70±2.45	7.22±5.91	4.57±1.27	7.78±3.96	0.108
BGT	156.80±66.97	158.11±56.35	155.43±43.81	125.33±47.69	0.267
Cst (mL/cmH_2_O)	32.88±10.48	30.55±11.06	30.84±9.48	25.17±7.90	0.392
Rrs (cmH_2_O/L/s)	13.65±5.11	12.33±1.73	13.14±5.27	15.00±6.74	0.727
HR (bpm)	81.70±18.80	88.33±19.71	89.14±17.02	85.56±12.75	0.799
SpO_2_ (%)	97.90±1.96	97.44±2.92	97.00±2.44	97.44±2.18	0.897
SBP (mmHg)	135.80±2727.21	124.44±22.73	134.29±27.43	136.11±20.44	0.712
DBP (mmHg)	77.90±16.01	69.33±9.31	83.86±13.85	76.56±16.06	0.253
Temperature (°C)	36.30±0.67	36.94±0.71	36.64±0.43	36.67±0.38	0.140
Reason for admission					
Respiratory disorder	4 (40.0)	5 (55.5)	3 (42.8)	4 (44.4)	-
Cardiac disorder	2 (20.0)	2 (22.2)	2 (28.5)	2 (22.2)	-
Infection	2 (20.0)	1 (11.1)	1 (14.2)	2 (22.2)	-
Others	2 (20.0)	1 (11.1)	1 (14.2)	1 (11.1)	-
Comorbidity condition					
Respiratory	2 (20.0)	2 (22.2)	1 (14.2)	1 (11.1)	-
Cardiac	3 (30.0)	1 (11.1)	2 (28.5)	3 (33.3)	-
Endocrine	1 (10.0)	1 (11.1)	1 (14.2)	1 (11.1)	-
Urinary	1 (10.0)	2 (22.2)	3 (42.8)	1 (11.1)	-
Chronic renal failure	2 (20.0)	2 (22.2)	1 (14.2)	2 (22.2)	-
Infection	1 (10.0)	3 (33.3)	1 (14.2)	1 (11.1)	-

Data are reported as absolute numbers (%) and means±SD. One-way analysis of variance was used for statistical analysis. BMI: body mass index; APACHE II: Acute Physiology and Chronic Health Evaluation; WB-24 h: 24-hour water balance; RASS: Richmond Agitation-Sedation Scale; T MV: time under mechanical ventilation before starting the study protocol; T ICU: time at the intensive care unit before starting the study protocol; BGT: blood glucose test values; Cst: static compliance of the respiratory system; Rrs: Resistance of the respiratory system; HR: heart rate; SpO_2_: peripheral oxygen saturation; SBP: systolic blood pressure; DBP: diastolic blood pressure; FES: functional electrical stimulation; PCE: cycle-ergometry passive.

Supplementary Table S1 reports the NO values cultured in monocytes stimulated (C+) and non-stimulated (C-) assessed before and 1 h after applying the study protocol to the four study groups. A significant decrease in NO production was noted in stimulated and non-stimulated cells comparing the results obtained before and after treatment in the FES and PCE groups.

Supplementary Table S2 demonstrates the analysis of TNF-α, IFN-γ, IL-6, and IL-10 levels before and after the intervention showing a significant decrease only in the TNF-α concentration in the PCE group.

## Discussion

To our knowledge, this is the first study to compare the effects of different types of exercise on nitrosative stress and inflammatory cytokines in critically ill patients. This study demonstrated that both PCE and FES reduced nitrosative stress, as shown by the reduction in cellular NO levels. PCE was the only therapy that reduced the concentration of TNF-α, thus decreasing the inflammatory process commonly observed in critically ill patients.

Few studies have been published describing the effects of FES and PCE on oxidative and nitrosative stress and inflammatory cytokines among critically ill patients (14,15). However, several additional benefits of these interventions were previously described, especially regarding the reduction in muscle mass loss and functional improvement (3).

Pro-inflammatory cytokines and oxidative and nitrosative stress have been investigated as potential causes of ICU-acquired muscle weakness; however, physical activity may modify the inflammatory response in this population (3). It should be noted that the immune response might vary with the intensity, duration, and frequency of exercise. Regular physical exercise is a therapeutic and/or preventive agent for numerous clinical conditions; however, acute physical exercise may be exhausting, also inducing important organic changes, particularly when the patients are not fully adapted to support the different loadings that are demanded. The magnitude of damage apparently depends on the intensity, duration, and type of exercise (16).

Given that oxidative and nitrosative stress is strongly associated with the pathophysiology of numerous diseases, reconciling the increase in oxidative and nitrosative stress induced by acute exercise with its potential beneficial effects may be difficult at first (17). However, when effectively adapted to each individual, the regular practice of physical exercise may increase the capacity of organic defense against the occurrence of these oxidative and nitrosative lesions in different organs and body tissues, thus increasing cellular and tissue protection in those individuals (18).

We observed a decrease in NO production in stimulated and non-stimulated cells when comparing the results obtained before and after FES and PCE. These results indicated that FES and PCE were separately effective in critically ill patient, positively changing the cellular redox status by decreasing the baseline levels of nitrogen reactive species one hour after the protocol. The duration and intensity level of the activity had a positive regulation of the antioxidant defense. Indeed, regular physical exercise results in antioxidant capacity adaptations, which protect cells against the deleterious effects of oxidative and nitrosative stress, thereby preventing subsequent cellular damage (19).

Gerovasili et al. (20) reported that the early application of FES to critically ill patients induces acute effects on systemic microcirculation. Those effects were demonstrated by the increase in thenar eminence muscle blood perfusion assessed using a near-infrared spectroscopy device immediately after applying FES for 45 min. The systemic effect of FES observed in this study was demonstrated by the improvement in tissue perfusion in muscle groups beyond those electrically stimulated, indicating the potential of this method to prevent polyneuromyopathy in critically ill patients. This hypothesis is in agreement with the above study (20) demonstrating the possible effect of FES on the release of anti-inflammatory mediators at the stimulated site, which may subsequently reach the bloodstream, increasing the perfusion of distant muscle groups.

These findings corroborate the study by Reese et al. (21), which assessed the effects of FES combined with a controlled diet on oxidative stress in patients with progressive multiple sclerosis. A decrease in oxidative stress and excitotoxicity was associated with large functional gains in these patients. Similarly, Bustamante et al. (22) studied the effects of magnetic stimulation of the quadriceps on oxidative stress in patients with severe chronic obstructive pulmonary disease (COPD) for 8 weeks. The therapy was well-tolerated and caused no increase in muscle oxidative stress.

Similar findings were reported by Mercken et al. (23) after assessing the effects of exercise in a cycle ergometer on oxidative stress before and 4 h after the exercise in patients with COPD subjected to an 8-week pulmonary rehabilitation program compared with healthy individuals. A decrease in reactive species of oxygen and nitrogen with a consequent reduction in DNA damage was observed.

Similar to oxidative and nitrosative stress, some selected cytokines also affect muscle dysfunction and degradation in critically ill patients. In a model for cachexia, Reid and Li (24) suggested a synergistic interaction between reactive species of oxygen and nitrogen and pro-inflammatory cytokines, such as TNF-α, perhaps indicating a pathologic positive feedback loop that reduces the regulation of damaged muscle tissue repair. Thus, the direct suppression of muscle activity in the presence of TNF-α and the decrease in repair or increase in apoptosis lead to muscle weakening mediated by these inflammatory cytokines.

The only change observed in inflammatory cytokines was the decrease in TNF-α in the group of patients subjected to cycle-ergometry. No changes in any other cytokine studied (IL-6, IFN-γ, and IL-10) were observed comparing the results obtained before and after the intervention in all groups. The intensity level and the duration of the exercise and cytokine kinetics may have determined the changes in concentration. Of all cytokines tested, TNF-α is the first to peak in plasma in the first hours after exercise (25). The variability of the evaluation time of the function of these cells after exercise, the level of individual physical fitness, and the different experimental protocols used may explain the differences in findings (26).

Muscle contraction induces the synthesis and release of IL-6 in plasma in large quantities, regardless of TNF-α production. During exercise, even low-intensity exercise, IL-6 is also synthesized and released in peritendon and adipose tissues (27). In addition to the pro-inflammatory properties, high levels of IL-6 stimulate the release of anti-inflammatory cytokines, including IL-10 and IL-1Rα, into plasma. Muscle-derived IL-6 reduces TNF-α production, disrupting muscle degradation by destroying myosin (3). Although no significant changes in the levels of IL-6 and IL-10 cytokines were observed in any group, the only group that exhibited an increase in their values after applying the study protocol was the PCE group, which may have contributed to the changes in the TNF-α concentration 60 min later. IL-6 and IL-10 concentrations may have changed in another period of our study that was not monitored.

Studies suggest that exercise results in anti-inflammatory activity via IL-10 and IL-6 induction and TNF-α and IL-1β inhibition. Physical exercise has been explored as a regulating agent in inflammation and muscle function (3). Amidei and Sole (14) assessed the effects of passive exercise for 20 min on the vital signs, presence of pain, and inflammatory cytokines of 30 critically ill patients under mechanical ventilation for up to 72 h. These parameters were assessed at three time points: before, immediately after, and 60 min after passive cycle ergometry. This intervention was well tolerated and reduced the presence of pain and IL-6 levels after 60 min of passive exercise, demonstrating the anticipated physiological benefits of exercise. The authors also reported that this significant decrease in IL-6 concentration from baseline to the end of the intervention combined with the lack of significant changes at the end of the rest period following the intervention supports the notion that passive exercise not only has no adverse effect on inflammation but may also be responsible for reduced IL-6 levels.

Although no changes were observed in any inflammatory cytokine studied after a single session of PCE and FES, we believe that applying these therapies may have beneficial effects on immune response parameters. Accordingly, Karavidas et al. (28) studied the application of FES to lower limbs of patients with severe heart disease for six weeks and observed that FES had a direct effect on endothelial function and peripheral markers of anti-inflammatory activation. Specifically, the authors noted decreased levels of TNF-α and IL-6 and improved blood flow in the brachial artery observed by Doppler ultrasound.

In another study, Akar et al. (15) studied patients with COPD under MV to assess the effects of a total of twenty sessions of active mobility and FES on the weaning process, hospital discharge, and parameters of inflammatory mediators. The results revealed a significant improvement in the peripheral muscle strength of lower limbs in the groups of patients subjected to FES and exercises as well as FES alone. Furthermore, IL-6 and IL-8 concentrations were reduced in the group of patients subjected to FES. This study presents positive points for being one of the first in the literature to evaluate inflammatory cytology in critically ill MV patients exposed to different early mobilization strategies. As a negative point, we would highlight the small sample size.

Based on the above findings, both PCE and FES applied to MV critically ill patients were insufficient to change the serum levels of cytokines in a single session. However, when assessing the effects of PCE and FES on nitrosative stress, the results revealed reduced NO concentrations one hour after using these therapies, thereby indicating that these therapies may reduce cellular nitrosative stress when applied separately.

Although we attempted to control our patients' data using randomization, one possible limitation of our study was higher NO baseline values among the four participating groups, although we had a greater reduction in NO in the PCE group. The reduction was also observed in the FES group that had the lowest baseline level of the four groups studied. These high basic values occurred after randomization and were not controlled, which may have influenced our results. Importantly, these therapies had some effect on NO reduction and were beneficial for the critically ill patient.

New research on these interventions should increase the variability in the assessment time of the function of these cells after physical exercise, the level of individual physical fitness of the patients before the critical illness, and the duration and intensity of the activity established in the various protocols towards reaching more conclusive results.

## Supplementary Material

Click here to view [pdf].
